# Using Technology to Promote Patient Engagement in Nutrition Care: A Feasibility Study

**DOI:** 10.3390/nu13020314

**Published:** 2021-01-22

**Authors:** Shelley Roberts, Wendy Chaboyer, Zane Hopper, Andrea P. Marshall

**Affiliations:** 1 School of Allied Health Sciences, Griffith University, Gold Coast, QLD 4222, Australia; 2 Menzies Health Institute Queensland, Griffith University, Gold Coast, QLD 4222, Australia; w.chaboyer@griffith.edu.au (W.C.); a.marshall@griffith.edu.au (A.P.M.); 3 Gold Coast Hospital and Health Service, Gold Coast, QLD 4215, Australia; zane.hopper@health.qld.gov.au; 4 School of Nursing and Midwifery, Griffith University, Gold Coast, QLD 4222, Australia

**Keywords:** health information technology, nutrition intervention, patient engagement, patient participation in care

## Abstract

Empowering patients to participate in nutrition care during hospitalisation may improve their dietary intakes and associated outcomes. This study tested the acceptability and feasibility of a technology-based intervention to engage hospital patients in nutrition care at a tertiary teaching hospital in Australia. The hospital used an electronic foodservice system (EFS), by which patients ordered meals via bedside computers. Adults at nutritional risk received the nutrition technology (NUTRI-TEC) intervention, involving nutrition assessment, education on nutrition requirements and training on using the EFS to enter food intakes and monitor nutrition goals. Acceptability was assessed using patient satisfaction and engagement surveys. Feasibility was assessed by evaluating the intervention delivery/fidelity and patient recruitment/retention. Patients’ dietary intakes were observed daily to indicate the intervention’s effects and assess the accuracy of the patient-recorded intakes. Descriptive and inferential statistics were used to analyse the data. Of the 71 patients recruited, 49 completed the study (55% male; median (IQR) age 71 (65–78) years; length of stay 10 (7–14) days). Patient satisfaction with NUTRI-TEC was high. Intervention delivery and fidelity targets were met but recruitment (≥50%) and retention (≥75%) targets were not; only 31% of patients agreed to participate and 69% completed the study (mostly due to unexpected/early discharge). Patient- and researcher-recorded dietary intakes correlated strongly, indicating patients can record food intakes accurately using technology. This study highlights the important role technology is likely to play in facilitating patient engagement and improving care during hospitalisation.

## 1. Introduction

Modern-day healthcare is evolving at a rapid rate. Technological advances are facilitating more accessible, safe, effective, efficient and sustainable health care delivery [1,2]; and importantly, more patient-centred care [3]. A key characteristic of patient-centred care is patient engagement or participation, which is defined as active involvement in one’s own health care, for example, through sharing/exchanging health information or engaging in intellectual and/or physical health care activities [4]. Patient engagement is a core aspect of safe and quality health care endorsed by the World Health Organization’s Patients for Patient Safety movement [5]. It is associated with improved clinical and functional outcomes, as well as increased patient safety and satisfaction with care [6,7]. Health information technology (HIT) has the ability to facilitate patient engagement by providing the resources and tools required for patients to play an active role in their health care [3,8]. While there has been wide adoption of HIT by individuals in the community (e.g., applications, programs and web-based tools for monitoring measurable health parameters or for implementing lifestyle changes), HIT adoption in hospital settings is lagging [9]. As more research on HIT use in clinical settings emerges, showing promise for improved patient and hospital outcomes, it is imperative that healthcare professionals and organisations not only embrace HIT but contribute to its development, implementation and evaluation.

Nutrition and dietetics is a field that may benefit from HIT [10]. Not only is nutrition an area in which patients feel comfortable participating [11], HIT may provide solutions to long-standing problems, such as hospital malnutrition. In a landmark 1974 paper, Butterworth proposed that iatrogenic malnutrition was one of the most serious nutritional issues of the time, despite “the technological advances that have been made” [12]. Almost five decades later, and after an explosion in HIT, malnutrition remains a significant clinical problem. It affects 20–50% of hospital patients worldwide [13,14] and results in poor patient outcomes, including increased risks of mortality [15], infections [16], pressure injury [17], reduced mobility and falls [18,19]. It also contributes to increased lengths of hospital stays, readmissions and hospital costs [15,20], placing a large burden on healthcare systems. Malnutrition can be prevented or corrected with adequate dietary intake; however, due to a complex mix of factors, achieving optimal nutrition intake among hospitalised patients is difficult [21]. One strategy showing promise is patient participation in their nutrition care, with preliminary research showing this is an effective strategy for improving the dietary intakes of hospitalised patients [22,23].

Our team previously developed a paper-based intervention that allowed hospitalised patients to participate in their nutrition care by self-monitoring their dietary intake and engaging in guided nutritional goal setting [23]. Others have found the enactment of self-regulatory behaviours, such as setting goals and monitoring food intake, were the best predictors of participants’ nutrition status [24]. Using an integrated knowledge translation (IKT) approach [25], guided by the Knowledge to Action cycle [26], our team adapted this intervention for delivery via HIT that was compatible with existing hospital systems and models of care. This intervention has the potential to improve the dietary intakes of hospitalised patients at risk of malnutrition by engaging them in their care. While previous studies have shown increased energy and protein intakes among patients participating in food intake monitoring and guided nutritional goal setting (using paper resources), none have evaluated this kind of intervention using technology as the medium for engagement.

## 2. Materials and Methods

### 2.1. Study Overview and Aims

The aim of this study was to evaluate the acceptability and feasibility of the HIT intervention NUTRI-TEC, which engages hospital patients in their nutrition care in order to improve dietary intakes. Acceptability was determined with patient satisfaction and engagement surveys, which were assessed against criteria on acceptability. Feasibility (for a larger trial) was assessed against criteria on intervention delivery and fidelity, as well as patient recruitment and retention. The accuracy of patient-recorded (vs. researcher-recorded) dietary intakes, along with an indication of the intervention’s effects on patients’ intakes, were also assessed to give further insights into the intervention. This study received ethical approval from the relevant hospital and university human research ethics committees.

Feasibility criteria included:

(a) ≥80% of patients receive the intervention within 24 h of enrolment (delivery);
(b) ≥80% of patients receive all intervention components as intended (fidelity);
(c) ≥50% of patients approached for recruitment agree to participate (recruitment);
(d) ≥75% of patients remain in the study long enough for adequate outcome data collection, i.e., three days of oral intake data (retention).

We determined that for feasibility, the intervention delivery and fidelity would have to be ≥80% to allow for intention-to-treat analyses in a larger trial. For recruitment and retention, 50% and 75% were selected, respectively, as we recognised that not all patients may want to participate in research while in hospital and that the intervention (being technology-based) may not appeal to some patients. The values selected are consistent with those developed for a similar randomised controlled feasibility study of a complex intervention to reduce frailty and falls among haemodialysis patients [27].

Acceptability was explored quantitatively via patient satisfaction and engagement surveys. The intervention was considered acceptable if ≥80% of patients answered “satisfied” or “very satisfied” to the patient satisfaction survey questions.

Finally, patients’ daily total energy and protein intakes were monitored to give an indication of the effect of the intervention over time and to assess the accuracy of patient-recorded dietary intakes (vs. those recorded by RAs).

### 2.2. Setting

This study was conducted in four wards (orthopaedic, renal, respiratory and vascular/medical) at a large tertiary university hospital in southeast Queensland, Australia. The hospital used an electronic foodservice system (EFS; Delegate Food Service system, version 12.10, Delegate Technology GmbH; Vienna, Austria), which was accessed by each patient through their bedside computer. Standard practice at the hospital involved patients using the EFS to order their meals. The EFS had the functionality for patients to view nutrition information, including energy in kilojoules (kJ) and protein in grams (g), for each item on the hospital menu. Patients could choose whether to “show” or “hide” this information when ordering meals.

### 2.3. Sample and Recruitment

Adult patients at nutritional risk, who met all inclusion and no exclusion criteria, were eligible to participate. Inclusion criteria: (a) adults able to provide informed consent (≥18 years old, cognitively intact), (b) able to communicate in English (verbally and in writing), (c) at risk of malnutrition (aged ≥65 years and/or score of ≥2 on the Malnutrition Screening Tool (MST)), and (d) expected length of stay of ≥4 days from recruitment. Exclusion criteria: (a) previous participation in the study, (b) palliative or dying patients, (c) patients not receiving nutrition orally, and (d) patients with a history of an eating disorder.

A research assistant (RA) conducted daily participant eligibility screening on each study ward for 12 weeks. Eligible patients were given a participant information form and verbal explanation of the study and those willing to participate provided written informed consent and were enrolled. Patients remained in the study for 14 days or until death or hospital discharge, whichever occurred first. As there is no specific sample size calculation for feasibility studies [28], a literature-informed, pragmatic approach was used to determine sample size, based on work from Hertzog (2008) and on a previous pilot study of a similar intervention [23]. From this, it was expected that around 60–80 patients would be recruited with the available resources.

### 2.4. Intervention

Each patient received standard care plus the intervention, which was delivered by an RA (who was an accredited dietitian) within 24 h of recruitment. In addition to standard care, the intervention included an initial session consisting of:

(a) A brief nutrition history (Subjective Global Assessment (SGA)) conducted by an RA;
(b) A brief lesson on the importance of meeting nutritional requirements in hospital;
(c) Training on how to use EFS to order meals, enter food intake after each meal and view and monitor nutritional goals.

**Figure 1:**
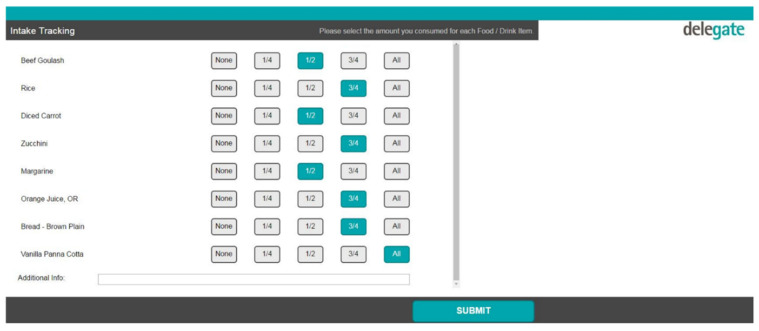
Intake tracking page in the electronic foodservice system (EFS).

**Figure 2:**
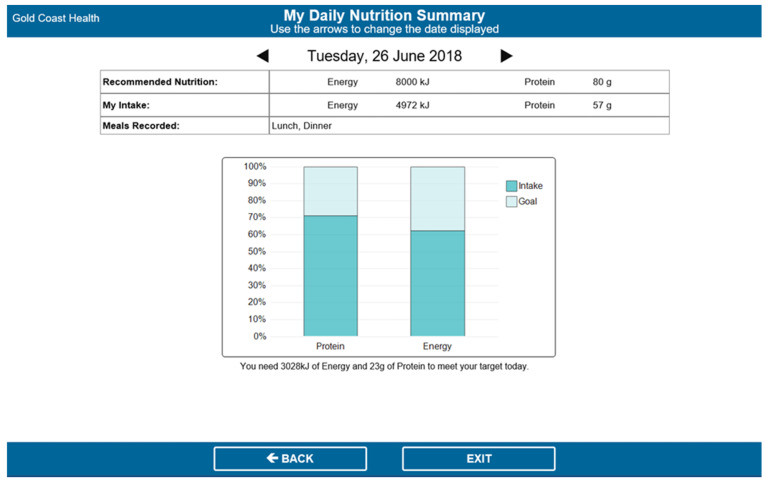
Nutrition goals page in the EFS.

After this initial session, patients were asked to record their food intake after each meal via the EFS. This involved opening the EFS portal from their bedside computer, selecting the day and meal (e.g., Monday breakfast) and selecting the amounts consumed for each meal item, presented in quartiles (see Figure 1). The EFS would only show the meal items ordered by and delivered to that patient on the intake tracking page. The EFS then calculated the patients’ energy (kJ) and protein (g) intakes for each meal and each day, and displayed this information on the nutrition goals page, alongside their individually estimated energy and protein requirements (see Figure 2).

The intervention also included review sessions conducted by RAs (daily for the first week after enrolment and every second day for the second week after enrolment) to engage patients in guided nutritional goal setting and monitoring via the EFS. The RAs discussed patients’ goals graphs with them and showed them how to use this information to inform their menu choices for the next day. If patients met <75% of their estimated energy/protein requirements on a given day, RAs reviewed possible strategies that patients could enact to help meet their requirements. A detailed description of intervention components and development can be found in a separate publication [29].

Clinically relevant data that were collected by the RAs (SGA score, patient preferences) were made available to ward dietitians, who prescribed nutrition support if required (e.g., oral nutrition supplements, high-energy/high-protein extras).

### 2.5. Data Collection

All data were collected by the same trained RAs and included the following:

Feasibility data pertaining to recruitment (number of patients recruited from those eligible), retention (number of patients remaining in the study until the end point) and intervention delivery (intervention components delivered and timing) were recorded by the RAs in standardised logs at patient recruitment, the study end point and upon intervention delivery, respectively.

Acceptability was assessed via a validated patient participation scale [7] and patient satisfaction survey [30], which was administered to patients by the RAs prior to discharge or on study day four, whichever came first.

Patient demographics (age, gender, admission details, diagnosis, co-morbidities) and nutrition data (weight, height, body mass index (BMI), MST and SGA scores, prescribed diet (including supplements), and dietitian input) were collected from patients’ electronic medical records and bedside charts after their recruitment. Patients’ individually estimated energy (EER) and protein requirements (EPR) were calculated by the RAs using local clinical guidelines [31] and entered into the EFS. Data on adverse events, including pressure injuries, falls and infections were collected from electronic medical records by the RAs upon participants’ hospital discharge.

Patients’ nutrition intakes were collected via visual observation of mealtimes and recorded until the study endpoint (i.e., 14 days, hospital discharge or death, whichever occurred first). Visual plate waste was observed by the RAs at each meal and the intake of each dietary item was recorded in quartiles on a standardised food chart, which is a method that has been shown to correlate with weighed food records [32]. Patients’ intakes were entered into a Foodworks database (version 9, Xyris Software; Brisbane, Australia) containing the nutritional information of dietary items provided by the hospital to calculate their daily energy and protein intakes. Intakes were presented as a proportion of EER and EPR for each patient. The amounts of energy and protein provided (i.e., food ordered by the patients) each day were exported from the EFS. If patients consumed <50% of their meal, the reasons for eating poorly were documented by observation (e.g., meal not delivered, patient not in bed) and/or by asking the patient (e.g., poor appetite, nutrition impacting symptoms, dislike of hospital food).

### 2.6. Data Analysis

Demographics, feasibility, nutrition intakes and survey data were analysed descriptively using SPSS Statistics for Windows version 23.0 (IBM Corp. 2012, Armonk, NY, USA). Continuous data were tested for normality using the Shapiro–Wilk test and presented accordingly (mean ± standard deviation for normally distributed data; median (interquartile range (IQR)) for non-normally distributed data). One-way repeated measures ANOVA tests were used to compare the proportion of EER and EPR met by patients each day over time. Patient-recorded intakes and researcher-observed intakes were compared using Pearson’s correlation to determine how accurately patients recorded their food intake. Significance was set to *p* ≤ 0.05.

## 3. Results

### 3.1. Participant Characteristics

**Figure 3:**
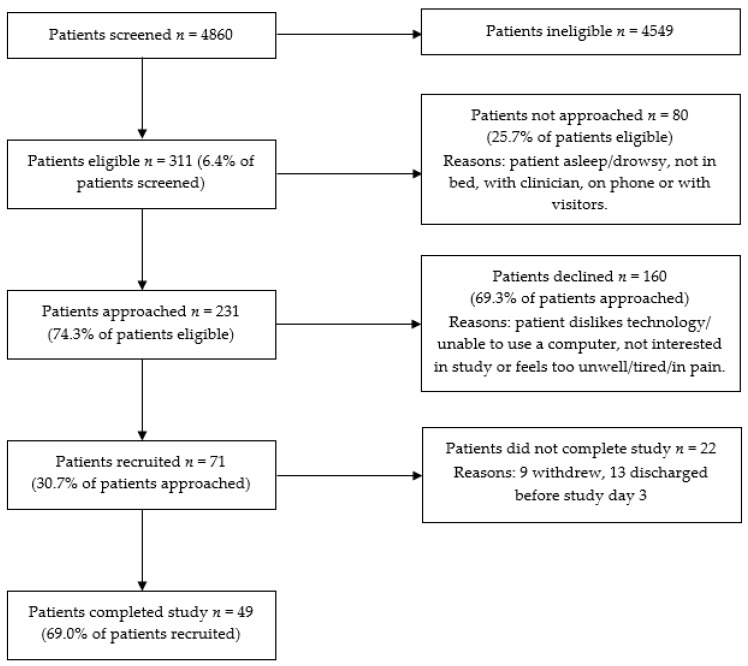
Participant flow diagram.

A total of 71 patients were recruited for the study (Figure 3). The characteristics of the total sample recruited (*n* = 71) and comparison between patients who did (*n* = 49) and did not (*n* = 22) complete the study are provided in Table 1. Significant differences were seen between patients who did and did not complete the study for age, study length of stay (LOS) and EER; there were no differences between groups for any other characteristics. The ages ranged from 27–89 years and patients were recruited from renal (34%), respiratory (30%), orthopaedic (21%) and vascular (15%) wards. Patients’ diagnoses were consistent with those typically found on the study wards, with respiratory (23%), orthopaedic (21%), cardiovascular (11%), renal (10%) and infectious (6%) conditions comprising the majority of diagnoses. Information on the nutrition care patients received can be found in Supplementary File 1.

### 3.2. Acceptability

#### 3.2.1. Patient Satisfaction

Of the 49 patients who completed the study, 36 completed patient satisfaction and participation surveys (Supplementary File 2). The remaining patients were discharged before the satisfaction survey could be administered. The patient satisfaction criterion was met, with 89% of patients satisfied with the nutrition care they received, 86% satisfied with the effect of their nutrition care and 94% satisfied with the explanations they were given regarding their nutrition.

#### 3.2.2. Patient Engagement

Results from the patient participation survey showed that patients were engaged in the intervention. Most (80%) said they participated in decisions about their nutrition to the extent they wanted to and 92% were aware of their nutrition plan. Nearly all patients (92%) agreed that the food and nutrition care they received was right for them and 91% reported receiving adequate help to meet their nutrition needs. Many patients (89%) said that it was easy to find information about their nutrition and 94% always felt well enough to discuss their nutrition with RAs. Seventy-two percent said that they knew a lot about their nutrition after the intervention and 94% understood the different menu options they had for meeting their nutrition goals. Around half of the patients (53%) reported that family members or friends also participated by helping to ensure their wishes were followed regarding food/nutrition.

### 3.3. Feasibility

#### 3.3.1. Intervention Delivery and Fidelity

The intervention delivery target of ≥80% was met; of the 49 patients who completed the study, all (100%) received the intervention and 40 (82%) received it within 24 h after enrolment. Nine patients received the intervention slightly late (within 24.5–28.5 h of enrolment) due to an overlap with the RAs’ daily screening/recruiting. The fidelity target of ≥80% was met; all 49 patients received at least one intervention component as intended. Most (*n* = 47; 96%) received nutrition history/SGA, education on meeting nutrition requirements in hospital and training on how to order meals via the EFS. Almost all patients (*n* = 48; 98%) received training on how to complete intake tracking and monitor nutrition goals. The median (IQR) time spent on the initial intervention delivery was 15 (10–20) minutes. The daily follow-up sessions’ completion was high (65–81%). Reasons that the follow-up sessions were not completed included patients being too unwell, nil by mouth, asleep, with clinicians or visitors, not in bed, or not interested. Mean (±SD) time spent delivering follow-up sessions was 7.9 (±3.7) minutes.

#### 3.3.2. Recruitment and Retention

The recruitment target (≥50%) was not met; only 31% of eligible patients who were approached agreed to participate in the study. As the recruitment rate was lower than expected, we tested for differences in recruitment rates between wards and RAs, which was not planned a priori. While there were no differences between wards, there were significant differences in recruitment rates between RAs, with more experienced RAs yielding higher rates of recruitment (*p* < 0.01). The retention target (≥75%) was also not met; only 69% of patients completed the study (i.e., had adequate outcome data collected for dietary intake analysis), mostly due to unexpected or early hospital discharges. The withdrawal rate was 13%.

### 3.4. Nutrition Intake Data

**Figure 4:**
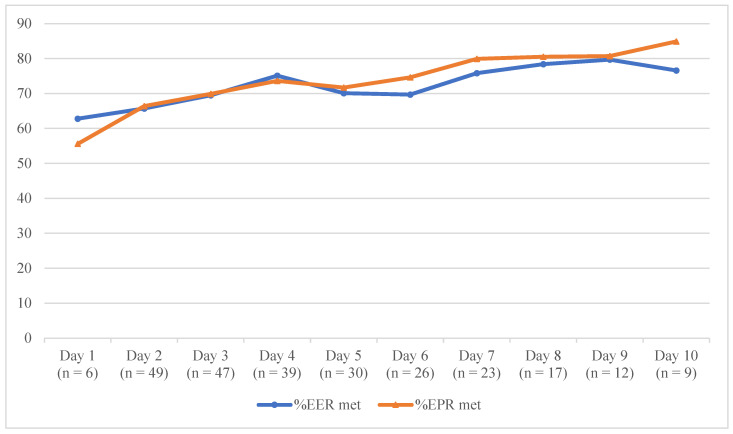
Mean proportion of patients’ estimated nutrition requirements met over time. %EER met: proportion of mean estimated energy requirements met; %EPR met: proportion of mean estimated protein requirements met.

The nutrition intakes of the 49 patients who completed the study (i.e., those who had adequate outcome data collected; that is, ≥3 days’ of oral intake data) over the study period are shown in Figure 4. Daily energy (kJ) and protein (g) intakes, and percentages of daily EER and EPR met over the 14 study days are included in Supplementary File 3. On average, participants consumed 66% of their EER and EPR on study day 2 (first day with complete nutrition intake data). This increased to 75% EER and 74% EPR on study day 4 (39 patients with complete intake data) and 80% EER and 81% EPR on study day 9 (12 patients with complete intake data). The patients’ self-reported dietary intakes correlated closely with intakes collected by the RAs (Pearson’s correlations were between 0.642 and 0.932 for all study days (*p* < 0.05), indicating strong correlations).

## 4. Discussion

This study evaluated the acceptability and feasibility of a HIT intervention that was designed to engage hospitalised patients in their nutrition care in order to improve their dietary intakes. This work provides important insights into the use of HIT for patient engagement in acute hospital settings where patient engagement in care is encouraged (and even mandated in some countries) and where HIT has the potential to revolutionise care provision and the patient experience.

This study found that the NUTRI-TEC intervention was acceptable to patients, with most (86–94%) reporting satisfaction with its various aspects. This is consistent with a qualitative study that explored patient perceptions of NUTRI-TEC among a subset of participants from the current study [33]. In the qualitative study, patients reported high satisfaction with NUTRI-TEC as they found it useful, valuable and easy to use, and they enjoyed gaining new knowledge and awareness regarding nutrition and participating in their nutrition care [33]. Patient satisfaction with health care interventions is necessary, but alone, it is insufficient for success; this requires meaningful patient engagement. Patients in this study had high levels of engagement with NUTRI-TEC. Over 90% of patients reported being aware of their nutrition plan, understanding their menu options and feeling well enough to discuss nutrition with the RAs; furthermore, 80% participated in decisions about their nutrition to the extent they wanted to. This is promising, as an integrative review on patient participation in acute medical wards found that engaging hospital patients in care is difficult, and patients’ expectations and desires for participation were often not met, with some participating more or less than they wished [34]. In our study, meaningful engagement in nutrition care (via NUTRI-TEC) involved patient education, i.e., learning about their nutrition needs and how to meet these needs in hospital; self-assessment and feedback, i.e., monitoring their nutrition intakes and requirements; behaviour change, i.e., being empowered with knowledge and control over their nutrition care, which allowed patients to make informed dietary choices. These activities align with a conceptual analysis of patient participation in care [4]. First, meaningful information exchange between patients and dietitians (in this case, RAs) occurred via data they each entered in the EFS, which both parties used to inform nutrition care. Dietitians/RAs surrendered some power/control, allowing patients to track their dietary intakes and make decisions about their nutrition. Using the EFS’s goals page in daily review sessions to guide discussions and monitor progress allowed for mutual involvement in health care activities and allowed patients and RAs to establish and build a relationship [4]. These concepts are of high importance for any patient engagement intervention and should be considered in both their development and delivery in practice.

The delivery of NUTRI-TEC was feasible using trained RAs, who were also dietitians. All patients received the intervention; most (82%) within 24 h and all within 29 h of recruitment. While nine patients received the intervention a few hours late due to an overlap with RA screening and recruiting, this problem would not occur in a larger trial as we would employ separate RAs for screening/recruiting, intervention delivery and data collection. Intervention fidelity was high, with >95% of patients receiving at least one component, and importantly, the intervention took only 15 min to deliver on average, which is critical for its feasibility in practice. NUTRI-TEC was designed to be used as part of routine clinical dietetic practice, which means these results are encouraging; however, as Rogers’ diffusion of innovations theory suggests, for uptake, innovations must demonstrate a relative advantage by being better than the idea/method they supersede [35]. NUTRI-TEC has a relative advantage over traditional methods of nutrition care, such as dietary intake monitoring, which is a core part of the nutrition care process. Nutrition intake data are vital for nutrition assessment and monitoring; however, commonly used methods of patient recall and nurse-recorded food charts are known to have low accuracy and reliability and are not always able to be achieved. One study found that only 64% of patients were able to complete a diet recall interview, which was time and resource intensive [36]. Another found that nurse-recorded food charts had low accuracy and completion, with 93% of patient intakes being unable to be determined due to missing data [37]. NUTRI-TEC allows patients (or their family members) to record food intakes, making it easier for dietitians to access dietary data to inform nutrition care, whilst engaging patients in their care. In early usability work, nurses at the study site indicated that they would prefer to enter patients’ intakes using NUTRI-TEC than a paper food chart if they were asked to by dietitians [38], suggesting that the intervention may also have a relative advantage for nurses. Furthermore, this study found that energy and protein intakes recorded by patients correlated strongly with those recorded by researchers, indicating that patients can accurately document their dietary intakes in the hospital using technology.

Despite promising findings on intervention delivery and fidelity, recruitment was challenging; only a third of eligible patients agreed to participate in the study. While this rate is suboptimal, it is not entirely surprising, given the intervention involved technology and participants were acutely unwell in hospital. Research shows that up to 75% of patients decline to participate in HIT studies, with a common reason being that participants perceive low benefits from technology [39]. Recruiting hospital patients to interventional research is also challenging; one review of fall prevention interventions reported a <50% median recruitment rate among institutionalised older patients [40]. Physical limitations, such as being tired or sick, are other frequently cited reasons for non-participation in clinical research [41]. Reasons for declining participation in the current study were consistent with previous work and included disinterest in, dislike of or unfamiliarity with technology, as well as feeling too unwell. However, there is scope for reducing such barriers. First, at the study hospital, some patients would have been unfamiliar with the EFS, as despite hospital policy, only around a third are shown how to use it on admission. This likely reduced some patients’ willingness to participate in a study involving this technology. Given all patients who participated in usability testing found NUTRI-TEC easy to use after a brief familiarisation and said they would use it in future admissions [11], further work could be done to improve patient orientation regarding the EFS. Second, for patients feeling too unwell to participate in NUTRI-TEC activities, there is potential for family members or hospital staff to participate on their behalf. For example, family members could enter patients’ food intakes and track their progress towards nutrition goals via the EFS, which anecdotally, some did in the current study. Allowing family members to participate on patients’ behalf could improve recruitment to a larger trial. Furthermore, family involvement in acute adult patients’ care has been shown to improve patient outcomes in some instances [42]. In practice, hospital staff, such as nurses or dietetic assistants, could record patient intakes (as they would with dietitian-requested food charts) via the EFS to begin with, then patients could take over this task as they start to improve/feel well. This may have an added benefit of patients feeling more confident completing intake tracking via the EFS after having watched someone else do it first.

The success of HIT interventions that aim to engage hospitalised patients in their care seems to depend on five key concepts that were identified in a realist review: information sharing, self-assessment and feedback, tailored education, user-centred design, and supporting patients to use HIT [43]. NUTRI-TEC employs all these concepts by engaging patients in dietary intake tracking (self-assessment/feedback) and nutritional goal setting (tailored education, information sharing) with RAs supporting patients in using and engaging with the intervention. NUTRI-TEC was underpinned by relevant theory and research frameworks, informed by literature and local data and iteratively developed and evaluated with end-user input for optimal success. Dietary data analysis in this study found that patients’ energy and protein intakes increased steadily over the first few days after receiving the intervention, which is consistent with our previous work. An earlier, paper version of this intervention was evaluated in a pilot randomised controlled trial (RCT) using paper food charts/goal pages, and found that while control patients’ intakes remained constant, intervention patients’ energy and protein intakes increased significantly following intervention delivery to a degree that was comparable to this study [23]. That previous pilot RCT found patients’ intakes increased from 65 to 83% of the EER being met and 65 to 91% of the EPR being met over the three days after intervention delivery, while the current study found increases in patients’ intakes from ~66% of the EER and EPR being met to ~75% of the EER and EPR being met over the same period. While this is a feasibility study, these results are promising, especially considering that research suggests patients that meet ≥75% of EER maintain their body weight during hospitalisation [32]. While patients’ intakes in this study did not increase as much as in the pilot RCT, the findings warrant further investigation.

### 4.1. Implications and Recommendations

NUTRI-TEC offers an alternative approach to patient education and counselling, dietary intake monitoring and nutrition care planning, and can potentially streamline nutrition care processes for dietitians. This novel HIT program allows dietitians to enter patients’ individually estimated energy and protein requirements, which patients can view at the bedside and dietitians can use as a tool for patient education (e.g., to guide discussions on dietary adequacy or nutrition plans). This approach is likely to result in more meaningful patient engagement, as patients will receive information/education that is individually tailored to them, which is a recognised feature of successful HIT interventions that aim to engage hospital patients in care [43]. It also allows patients to be more aware/informed of their nutrition care plan, which may improve adherence to nutrition interventions, as patients will better understand why these are needed. Using NUTRI-TEC as a patient education tool caters to different learning styles, with visual (graphical and numerical) and verbal (dietitian explanations) information available. This is important considering hospital patients may have low health literacy, particularly when it comes to accessing, appraising and understanding health information [44]. Nutrition interventions employing HIT have unbounded potential to improve nutrition care and dietary intakes and increase consumer engagement in hospital patients. However, as this study shows, HIT interventions, such as NUTRI-TEC, may not appeal to all patients, even if they are developed in a patient-centred way. Clinicians must therefore consider patients’ needs/preferences and tailor interventions accordingly to optimise patient uptake and clinical effectiveness. For patients wanting to engage in HIT interventions, clinicians must ensure the technology is interactive and easy to use and be willing to support/assist patients in their use. Lastly, as hospitalisation reflects a very short period in a patient’s life, it may not be the best place to deliver nutrition interventions; in this study, 18% of patients were discharged early and hence did not complete the study. Alternative methods for delivering and assessing the effects of interventions are therefore warranted, i.e., for patients with shorter lengths of stay. This may involve initial intervention delivery in hospital, followed by continued patient engagement after discharge (e.g., via an app or web-based program that patients can access at home that enables information exchange with dietitians) to allow for ongoing education and dietary intake and goal monitoring, as well as longer-term outcomes assessment. This could reduce the burden on hospital clinicians (in intervention delivery) and patients (by participating in interventions at home rather than when they are acutely unwell) and could help to address the notoriously poor follow-up of patients’ nutrition in the community. While the task of designing and implementing an intervention that bridges the hospital-to-home transition may be challenging, HIT could be the answer to improving the continuity of nutrition care in this population.

### 4.2. Limitations

This study has some limitations. First, it was a single-site feasibility study, which means that results may not be generalisable to other hospitals; however, it does provide important insights into conducting interventional HIT research with patients in hospital settings. Second, the low consent rate resulted in the recruitment target not being met; however, by collecting a range of feasibility data, we were able to identify that recruitment methods need revising for a larger trial (the primary purpose of feasibility studies). The low consent rate may have also resulted in a selection bias; hence, some of the positive findings (e.g., patient satisfaction) may have been overestimated. The 13% drop-out rate could be an indication that the intervention was not acceptable to a proportion of patients and should be considered when interpreting findings. A major limitation of the intervention itself is that only selected hospitals in Australia have electronic foodservice systems and patient bedside computers; both of which are needed for intervention delivery and engagement. However, with the expansion of HIT in hospitals and health care institutions, it is likely that this will be more widely available in the future. There may also be alternative ways to deliver such an intervention, for example, with patients’ personal devices. Lastly, the NUTRI-TEC intervention focuses on improving patients’ dietary intakes in hospital; however, hospitalisation is a relatively short period in a person’s life. Patients would benefit from ongoing nutrition support that provides continuity through care transitions (e.g., hospital to home); hence, further work should be done to achieve this with HIT.

## 5. Conclusions

This study found high patient acceptability, satisfaction and engagement with the NUTRI-TEC intervention, which allows patients to self-monitor their energy and protein intakes relative to their energy/protein requirements from the hospital bedside. The intervention was feasible and efficient to deliver; however, recruitment and retention were challenging. The low consent rate (31%) and sizeable withdrawal rate (13%) may suggest that the intervention is not suitable for all hospital patients; hence, it should be targeted at those who are most likely to benefit from it. Patients were able to accurately record their energy and protein intakes using HIT and there was a trend for improved dietary intakes in the days following intervention delivery. Prior to conducting a randomised controlled trial to evaluate the intervention’s effects on patients’ dietary intakes and nutrition-related outcomes, further work will be done to improve recruitment and optimise retention. We will also consider alternative ways for patients to access the intervention (e.g., through personal devices), both in hospital and after discharge, so we can investigate how nutrition care can be made more consistent, especially across care transitions in future work. This study highlights the important role HIT may have in the care of hospital patients in the near future, with a focus on patient engagement and participation.

## Figures and Tables

**Table 1:** Participant characteristics.

Characteristics	All Patients Recruited (n = 71)	Patients Who Completed Study (n = 49) *	Patients Who Did Not Complete the Study (n = 22) ^	p-Value
Female, n (%)	35 (49.3)	22 (44.9)	13 (59.1)	0.269
Age (years) ^a^	72.0 (66.0–80.0)	71.0 (64.5–77.5)	74.5 (68.8–84.0)	0.048
Hospital LOS (days) ^a^	9 (6–16)	10 (7–14)	9 (4–18)	0.227
Study LOS (days) ^a^	5 (3–9)	7 (5–10)	2 (1–3)	<0.001
ICU admission, n (%)	8 (11.3)	5 (10.2)	3 (13.6)	0.672
BMI (kg/m^2^) ^b^	26.8 ± 6.8	27.7 ± 7.6	24.9 ± 4.0	0.192
EER ^b^	9317 ± 1889	9584 ± 1881	8545 ± 1740	0.050
EPR ^b^	92.3 ± 18.8	92.6 ± 19.1	91.6 ± 18.6	0.851
SGA score ^c^, n (%)				
A	43 (65.2)	31 (63.3)	12 (70.6)	0.545
B	21 (31.8)	17 (34.7)	4 (23.5)	
C	2 (3.0)	1 (2.0)	1 (5.9)	

BMI: body mass index; ICU: intensive care unit; EER: estimated energy requirements; LOS: length of stay; EPR: estimated protein requirements; SGA: subjective global assessment; ^a^ presented as median (IQR) as data not normally distributed; ^b^ presented as mean ± SD (data normally distributed); ^c^ SGA scores: A (well-nourished), B (mildly–moderately malnourished), C (severely malnourished); * indicates participants who were included in data analysis (i.e., who completed the study); ^ indicates participants who were not included in the data analysis (i.e., who did not complete study).

## Data Availability

The data presented in this study are available on request from the corresponding author. The data are not publicly available as this was not approved in the ethics application.

## Supplementary Materials

The following are available online at https://www.mdpi.com/2072-6643/13/2/314/s1, Supplementary File 1, Nutrition care data; Supplementary File 2, Patient participation and satisfaction survey data; Supplementary File 3, Nutrition intake data.
