# Supplementary material for: Using Technology to Promote Patient Engagement in Nutrition Care: A Feasibility Study
Source: Nutrients. 2021 Jan 22;13(2):314. doi: 10.3390/nu13020314 (PMC7910973; doi:10.3390/nu13020314)
Supplement: Supplementary file 1 [file nutrients-13-00314-s001.pdf]

Supplementary File 1: Nutrition care data

Malnutrition risk screening (using the Malnutrition Screening Tool; MST) was not consistently completed in patients' charts by nurses; 32 (45%) patients' MSTs were completed, 30 (42%) were not completed, and nine (13%) were partially completed. Scores documented by nurses did not correspond with scores obtained by RAs (who were trained dietitians) in 71% of cases (as per table S1). MST scores documented by nurses tended to underestimate patients' actual MST scores.

Table S1. MST scores as completed by nurses vs. RAs.

MST score	Completed by nurse	Completed by RA	*p* value
0	27 (64.3%)	16 (24.2%)	0.049[a]
1	2 (4.8%)	19 (28.8%)	
2	9 (21.4%)	22 (33.3%)	
3	4 (9.5%)	8 (12.1%)	
4	0 (0%)	1 (1.5%)	
5	0 (9%)	0 (0%)	

[a]As per Pearson's Chi-Square test.

Of the total 71 patients recruited, 30 (42%) were seen by a dietitian during their admission (no difference between patients who did/did not complete study). Patients were typically prescribed full (45%), high protein (31%) or diabetic (16%) diets. Only 14 patients (20%) were prescribed supplements during their hospital stay (on average, these supplements provided 2305±1202kJ and 30±10g protein per day).

Supplementary File 2: Patient participation and satisfaction survey responses (*n*=36)

Satisfaction questions	Very satisfied	Satisfied	Neither satisfied nor dissatisfied	Dissatisfied
Are you satisfied with the care you received in hospital?	25 (69.4%)	7 (19.4%)	2 (5.6%)	2 (5.6%)
How satisfied are you with the effect of your nutrition care?	16 (44.4%)	15 (41.7%)	4 (11.1%)	1 (2.8%)
How satisfied are you with the explanations you were given about your nutrition?	21 (58.3%)	13 (36.1%)	2 (5.6%)	0

Participation questions	Strongly agree	Agree	Disagree	Strongly disagree	N/A
I participated in decisions about my nutrition (i.e., foods ordered) to the extent I wanted to	11 (30.6%)	17 (47.2%)	7 (19.4%)	0	1 (2.8%)
I was aware of my nutrition plan	9 (25.0%)	24 (66.7%)	3 (8.3%)	0	-

The food and nutrition care I received was right for me	14 (38.9%)	19 (52.8%)	3 (8.3%)	0	-
I received adequate help to meet my nutrition needs	18 (50.0%)	14 (38.9%)	3 (8.3%)	0	1 (2.8%)
It was easy to find information about my nutrition	13 (36.1%)	19 (52.8%)	4 (11.1%)	0	
I always felt well enough to be able to talk about nutrition	13 (36.1%)	19 (52.8%)	2 (5.6%)	0	2 (5.6%)
I know a lot about my nutrition (food intake vs. nutrition goals)	6 (16.7%)	20 (55.6%)	10 (27.8%)	0	-
I understood the different options for meeting my nutrition goals (e.g., different menu options)	10 (27.8%)	23 (63.9%)	2 (5.6%)	0	1 (2.8%)
Family members or friends helped me make sure my wishes were being followed (around food and nutrition)	10 (27.8%)	9 (25.0%)	7 (19.4%)	2 (5.6%)	8 (22.2%)

Supplementary File 3: Patients' nutrition intakes over time

Study day	Number of patients (*n*)		Nutrition intake data[a]			
	**Remaining in study**	**With complete intake data[c]**	**Energy intake (kJ)**	**EER met (%)**	**Protein intake (g)**	**EPR met (%)**
1	49	6[b]	6568 (±1920)	62.8 (±24.7)	65.5 (±33.7)	55.6 (±27.5)
2	49	49	6604 (±3004)	65.7 (±26.6)	65.7 (±31.6)	66.4 (±28.8)
3	49	47	7369 (±3650)	69.5 (±26.4)	77.2 (±45.4)	69.9 (±29.5)
4	49	39	7935 (±3099)	75.1 (±24.0)	79.6 (±36.2)	73.6 (±25.9)
5	39	30	6875 (±2512)	70.1 (±22.6)	69.5 (±28.9)	71.7 (±24.2)
6	32	26	7180 (±3486)	69.7 (±28.0)	77.8 (±39.6)	74.6 (±24.6)
7	27	23	7773 (±2843)	75.8 (±23.2)	82.2 (±26.8)	79.9 (±18.5)
8	23	17	8402 (±3376)	78.4 (±19.2)	86.3 (±33.1)	80.5 (±18.9)
9	18	12	9222 (±3657)	79.7 (±27.1)	92.4 (±39.6)	80.7 (±26.4)
10	13	9	8937 (±3734)	76.6 (±23.1)	93.5 (±36.1)	84.9 (±17.8)
11	9	6	8525 (±5006)	71.1 (±24.2)	96.8 (±68.1)	81.0 (±16.1)
12	5	5	9035 (±3824)	79.0 (±25.8)	93.6 (±44.3)	81.5 (±22.2)
13	5	4	8777 (±3113)	79.7 (±25.8))	80.8 (±23.8)	85.7 (±17.7)
14	4	2	9868 (±1179)	90.3 (±12.4)	115.1 (±16.7)	100 (0)

[a]As the majority of data were normally distributed (as per Kolmogorov-Smirnov test), data are represented as mean (±SD). [b]Only six patients had a complete day of nutrition intake data collected on study day 1, as most patients were recruited after breakfast and not all meals were observed. [c]Patients with incomplete nutrition intake data on any given day (i.e., patient was fasting or missed a meal due to a procedure or being on day leave, RA missed a meal observation, or patient was discharged) were excluded from analyses on that day. EER: Estimated energy requirements
EPR: Estimated protein requirements.
